# Single nucleotide variants in microRNA biosynthesis genes in Mexican individuals

**DOI:** 10.3389/fgene.2023.1022912

**Published:** 2023-03-02

**Authors:** Jesús Juárez-Luis, Moisés Canseco-Ocaña, Miguel Angel Cid-Soto, Xochitl H. Castro-Martínez, Angélica Martínez-Hernández, Lorena Orozco, Araceli Hernández-Zavala, Emilio J. Córdova

**Affiliations:** ^1^ Section of Research and Postgraduate, Superior School of Medicine, National Institute Polytechnique, Mexico City, Mexico; ^2^ Oncogenomics Consortium Laboratory, National Institute of Genomic Medicine, Mexico City, Mexico; ^3^ Genomics of Psychiatric and Neurogenerative diseases Laboratory, National Institute of Genomic Medicine, Mexico City, Mexico; ^4^ Immunogenomics and Metabolic diseases Laboratory, National Institute of Genomic Medicine, Mexico City, Mexico

**Keywords:** miRNA, SNV, biosynthesis of miRNAs, Mexican, genotyping

## Abstract

**Background:** MicroRNAs (miRNAs) are important regulators in a variety of biological processes, and their dysregulation is associated with multiple human diseases. Single nucleotide variants (SNVs) in genes involved in the processing of microRNAs may alter miRNA regulation and could present high allele heterogeneity in populations from different ethnic groups. Thus, the aim of this study was to genotype 15 SNVs in eight genes involved in the miRNA processing pathway in Mexican individuals and compare their frequencies across 21 populations from five continental groups.

**Methods:** Genomic DNA was obtained from 399 healthy Mexican individuals. SNVs in *AGO2* (rs2293939 and rs4961280), *DGCR8* (rs720012), *DICER* (rs3742330 and rs13078), *DROSHA* (rs10719 and rs6877842), *GEMIN3* (rs197388 and rs197414), *GEMIN4* (rs7813, rs2740349, and rs4968104), *TNRC6B* (rs9611280), and *XP05* (rs11077 and rs34324334) were genotyped using TaqMan probes. The minor allele frequency of each SNV was compared to those reported in the 1,000 Genomes database using chi-squared. Sankey plot was created in the SankeyMATIC package to visualize the frequency range of each variant in the different countries analyzed.

**Results:** In Mexican individuals, all 15 SNVs were found in Hardy-Weinberg equilibrium, with frequencies ranging from 0.04 to 0.45. The SNVs rs4961280, rs2740349, rs34324334, and rs720012 in Mexican individuals had the highest minor allele frequencies worldwide, whereas the minor allele frequencies of rs197388, rs10719, rs197414, and rs1107 were among the lowest in Mexican individuals. The variants had high allele heterogeneity among the sub-continental populations, ranging from monomorphic, as was the case for rs9611280 and rs34324334 in African groups, to >0.50, which was the case for variants rs11077 and rs10719 in most of the populations. Importantly, the variants rs197388, rs720012, and rs197414 had F_ST_ values > 0.18, indicating a directional selective process. Finally, the SNVs rs13078 and rs10719 significantly correlated with both latitude and longitude.

**Conclusion:** These data indicate the presence of high allelic heterogeneity in the worldwide distribution of the frequency of SNVs located in components of the miRNA processing pathway, which could modify the genetic susceptibility associated with human diseases in populations with different ancestry.

## Introduction

MicroRNAs (miRNAs) are small non-coding RNAs approximately 22 nucleotides in length that have important functions in the post-transcriptional regulation of gene expression (López-Jiménez and Andrés-León, 2021). Most miRNAs bind to the 3′UTR region of their target messenger RNA, promoting either its degradation or the repression of its translation (Annese et al., 2020). Novel functions of miRNAs in promoting transcription and enhancing translation through their binding to the 5′UTR or promoter regions have also been demonstrated recently (Younger et al., 2009). MiRNA encoding genes account for ∼3% of the human genome and at least 30% of the protein-coding genes have been estimated to be regulated by miRNAs (Ni and Leng, 2015; Stavast and Erkeland, 2019).

MiRNAs are important regulators of a vast number of cellular processes, including proliferation, differentiation, intracellular signaling, and metabolism. Accordingly, alterations in the regulation of miRNAs are common in a variety of human diseases. For example, aberrant expression of miRNAs has been observed in different types of cancer, including lung, gastric, breast, and hepatocellular carcinoma (Volinia et al., 2006; Roth et al., 2011; Morishita et al., 2016; Kandettu et al., 2020). Similarly, alterations in the expression of miRNAs have been found in diabetes, cardiovascular diseases, and neuro-degenerative illnesses (Margis et al., 2011; Hashimoto and Tanaka, 2017; Yan et al., 2019).

The miRNA biogenesis pathway is a strictly regulated process composed of several enzymatic steps, starting with their transcription by RNA polymerase II as a long (∼1 Kb) primary hairpin structure, primary miRNA (pri-miRNA). The pri-miRNAs are processed in the nucleus by a protein complex composes of the ribonuclease (RNAse) III DROSHA and DiGeorge Syndrome Critical Region 8 protein (DGCR8) into a precursor miRNA (pre-miRNAs) of ∼65–70 pb. Next, the pre-miRNAs are exported to the cytoplasm through the Exportin-5 (XPO5) RAN-GTP complex, where they are cleaved by DICER, a second RNAse III protein, in complex with the trans-activating response RNA binding protein (TRBP). This processing step produces an ∼22 bp mature duplex composed of a guide miRNA strand and passenger *miRNA. The guide strand is selected and loaded into the RNA-induced silencing complex (RISC) by the Argonaute proteins (AGO1-4), whereas the *miRNA is usually degraded. The proteins GEMIN3 and GEMIN4 complete the formation and activity of the RISC (Saliminejad et al., 2019).

Recent studies have highlighted an association between single nucleotide variants (SNVs) in the miRNA processing genes and different human diseases. For example, SNVs rs197414 in *GEMIN3*, rs3742330 in *DICER1,* rs7813 in *GEMIN4,* and rs11077 in *XP05* have been associated with increased susceptibility to bladder, colorectal, gastric, and thyroid carcinoma, respectively (Yang et al., 2008; Xie et al., 2015; Zhao et al., 2015; Wen et al., 2017). SNVs in genes involved in the miRNA biogenesis pathway have also been associated with non-malignant diseases. For example, the SNVs rs13078 in *DICER*, rs10719 in *DROSHA,* and rs720012 in *DGCR8* have been associated with type 2 diabetes, primary hypertension, and late onset pre-eclampsia (Zhang et al., 2017; Huang et al., 2019a; Wen et al., 2019), respectively.

Previous studies have demonstrated that the frequency of the SNVs and strength of their association with human diseases are strongly dependent on ethnicity (Mallick et al., 2016; Gurdasani et al., 2019; Gnagnarella et al., 2021). In this sense, the minor allele frequency (MAF) of SNVs rs2237897 and rs2237892 in *KCNQ1* is significantly higher in East Asian populations (0.39 and 0.38, respectively) compared to European-derived groups (0.04 and 0.06, respectively), and these SNVs have been associated with type 2 diabetes mellitus in Japanese individuals but not in European subjects (Unoki et al., 2008; Yasuda et al., 2008; Rosenberg et al., 2010). Similarly, 11 variants in the HBA1/2 locus were specifically associated with red blood cell traits in individuals with African or Amerindian ancestries, but not in European-derived populations (Hodonsky et al., 2020). Thus, genetic associations previously observed in populations with European, Asian, or African ancestry may be different in populations with high admixture levels.

The current population in Mexico is mainly composed of a recent admixture of original Amerindian (56%), European (41%) and, to a lesser extent, African individuals (3%) (Moreno-Estrada et al., 2014). The complex admixture present in the Mexican population may substantially affect the frequency of variants occurring in genes involved in the miRNA biosynthesis pathway. Therefore, the present study aimed to determine the frequency of 15 variants in genes from the miRNA machinery pathway. The frequencies of these variants in Mexican individuals were also compared to those reported by the 1,000 Genome project for 21 different populations across the world.

## Materials and methods

### Study population

The sample population included 399 non-related healthy volunteers, 122 men (30.6%) and 277 women (69.4%), with a mean age of 43 ± 9.1 years. All participants were Mexican individuals with parents and grandparents born in Mexico and were recruited from four different geographical regions in the country: north (*n* = 100), central east (*n* = 100), south (*n* = 100) and south east (*n* = 99). Geographical regions were previously described elsewhere (Moreno-Estrada et al., 2014). Each participant signed a letter of informed consent. This study was carried out according to the Declaration of Helsinki and approved by the ethics and research committees of the National Institute of Genomic Medicine at Mexico City. In addition, we included genotype data from 21 different subpopulations belonging to five continental populations: African [Esan in Nigeria (ESN); Gambian in Western Division, Gambia (GWD); Luhya in Webuye, Kenya (LWK); Mende in Sierra Leone (MSL); and Yoruba in Ibadan, Nigeria (YRI)], Admixed Latino American [African Caribbean in Barbados (ACB); Colombian in Medellin, Colombia (CLM); Mexican ancestry in Los Angeles, California (MXL); Peruvian in Lima, Peru (PEL); and Puerto Rican in Puerto Rico (PUR)], East Asian [Chinese Dai in Xishuangbanna, China (CDX); Han Chinese in Beijing, China (CHB); Japanese in Tokyo, Japan (JPT); and Kinh in Ho Chi Minh City, Vietnam (KHV)], European [Utah residents with Northern and Western European ancestry (CEU); Finnish in Finland (FIN); British in England and Scotland (GBR); Iberian populations in Spain (IBS); and Toscani in Italy (TSI)], South Asian [Bengali in Bangladesh (BEB) and Punjabi in Lahore, Pakistan (PJL)] (Supplementary Table S1). Genotype data from all 21 subpopulations were incorporated from the International Genome Sample Resource (IGSR) (Clarke et al., 2017).

### Selection of gene variants

Fifteen SNVs in eight genes involved in the miRNA biosynthesis pathway (*AGO2*: rs2293939, rs4961280; *DGCR8*: rs720012; *DICER*: rs3742330, rs13078; *DROSHA*: rs10719, rs6877842; *GEMIN3*: rs197388, rs197414; *GEMIN4*: rs7813, rs2740349, rs4968104; *TNRC6B*: rs9611280; *XP05*: rs11077, rs34324334; Table 1) were selected from a literature search conducted in the electronic database PubMed. All variants were selected based on previous association with human diseases. The MAF of these variants was >1% in the global population according to the 1,000 Genome project database (Clarke et al., 2017).

**TABLE 1:** Single nucleotide variants in the miRNA biosynthesis machinery in Mexican individuals.

Gene	Chr:Position (GRCh38)	SNV ID	Annotation	Alleles	MAF
***AGO2***	8:140541308	rs2293939	Synonymous	G/A	0.22
	8:140637315	rs4961280	Promoter	C/A	0.37
***DGCR8***	22:20111059	rs720012	3′UTR	G/A	0.45
***DICER1***	14:95087025	rs3742330	3′UTR	A/G	0.18
	14:95090410	rs13078	3′UTR	T/A	0.10
***DROSHA***	5:31401340	rs10719	3′UTR	G/A	0.35
	5:31532531	rs6877842	5′UTR	G/C	0.12
***GEMIN3***	1:111754860	rs197388	Promoter	A/T	0.07
	1:111766501	rs197414	Missense	C/A	0.05
***GEMIN4***	17:744946	rs7813	Missense	A/G	0.28
	17:745258	rs2740349	Missense	T/C	0.19
	17:746265	rs4968104	Missense	T/A	0.09
***TNRC6B***	22:40156115	rs9611280	Missense	G/A	0.04
***XPO5***	6:43523209	rs11077	3′UTR	T/G	0.37
	6:43567281	rs34324334	Missense	C/T	0.07

GRCh38, Genome Reference Consortium; MAF, minor allele frequency; SNV, single nucleotide variant.

### Sample genotyping

Genomic DNA was isolated from 10 ml of whole blood samples using the QIAamp DNA Blood Maxi kit (Qiagen, Valencia CA, United States) following the manufacturer’s protocol. Select SNVs were genotyped using TaqMan exonuclease assays on a QuantStudio 7 Flex Real-Time PCR (Applied Biosystems, Foster City, CA, United States). The genotyping call rate exceeded 95% for all SNVs. Genotype validation was performed by directly sequencing a random number of samples. One hundred percent concordance was found.

### Population differentiation analysis

We measured the level of population differentiation with the Wright’s fixation index (F_ST_) for each pair and for all populations, including our sample population of Mexican Mestizos, using Arlequin Software version 3.5.2.2 (Excoffier and Lischer, 2010). F_ST_ plots were created using the Lattice package in statistical environment R.

### Correlation of gene variants with geographical characteristics

To analyze the geographical distribution of the gene variants, the correlation coefficient was estimated between the MAFs of all the variants and the latitude and longitude coordinates from each included population. Correlation was evaluated by the Pearson’s test using R version 3.4.4 statistical software. *p* ≤ 0.05 was considered significant.

### Statistical analysis

Genotyping data were reported as frequencies. The Hardy-Weinberg equilibrium was evaluated using χ^2^ (Genepop version 4.7; 31). The MAF of each SNV was compared to those reported in the 1,000 Genomes database (Clarke et al., 2017) using χ^2^ (Genepop version 4.7; 31). Significant differences were established at *p* ≤ 0.05. Sankey plot was created using the package SankeyMATIC in R software and show the relationship between the frequency of each variant and the number of countries that present the same frequency range.

## Results

### Allele frequencies of SNVs in the miRNA biogenesis pathway in Mexican individuals

Based on previous reports in the literature, 15 SNVs in eight genes involved in the biosynthesis of miRNAs (*AGO2*: rs2293939 and rs4961280; *DGCR8*: rs720012; *DICER1*: rs3742330 and rs13078; *DROSHA*: rs10719 and rs6877842; *GEMIN3*: rs197388 and rs197414; *GEMIN4*: rs7813, rs2740349, and rs4968104; *TNRC6B*: rs9611280; and *XPO5*: rs11077 and rs34324334) were selected for genotyping in a sample of Mexican individuals. Of these 15 SNVs, 7 were located in coding regions (6 missenses, 1 synonymous), 5 in 3′UTRs, 2 in promoters, and 1 in 5′UTRs (Table 1).

After genotyping the DNA samples from 399 healthy individuals, the analyzed SNVs showed no significant deviation from Hardy-Weinberg equilibrium (*p* > 0.05). The minor allele of SNV rs9611280 in *TNRC6B* showed the lowest frequency (0.04), whereas variant rs720012 in *DGCR8* was the most frequent (0.45). The frequency of variants rs197388 and rs197414 in *GEMIN3*, rs34324334 in *XPO5,* rs4968104 in *GEMIN4*, and rs13078 in *DICER1* ranged from 0.05 to 0.10, whereas rs6877842 in *DROSHA*, rs3742330 in *DICER1*, and rs2740349 in *GEMIN4* presented frequencies between 0.12 and 0.19. Finally, variants rs2293939 and rs4961280 in *AGO2*, rs7813 in *GEMIN4*, rs10719 in *DROSHA*, and rs11077 in *XPO5* presented frequencies ranging from 0.22 to 0.37 (Table 1; Supplementary Table S2). We also performed analysis of haplotypes for all variants located in the same gene (rs2293939 and rs4961280 in *AGO2*, rs3742330 and rs13078 in *DICER1*, rs10719 and rs687782 in *DROSHA*, rs197388 and rs197414 in *GEMIN3*, rs7813, rs2740349, and rs4968104 in *GEMIN4* as well as rs11077 and rs343243343 in *XP05*). However, the linkage disequilibrium observed for each analyzed pairwise variants was below the threshold proposed by Gabriel et al. (2002) to consider a pair of SNVs as a haplotype, indicating that none of these variants form part of the same haplotype block.

### Allele frequencies of SNVs in Mexican individuals and other ethnic groups

**FIGURE 1:**
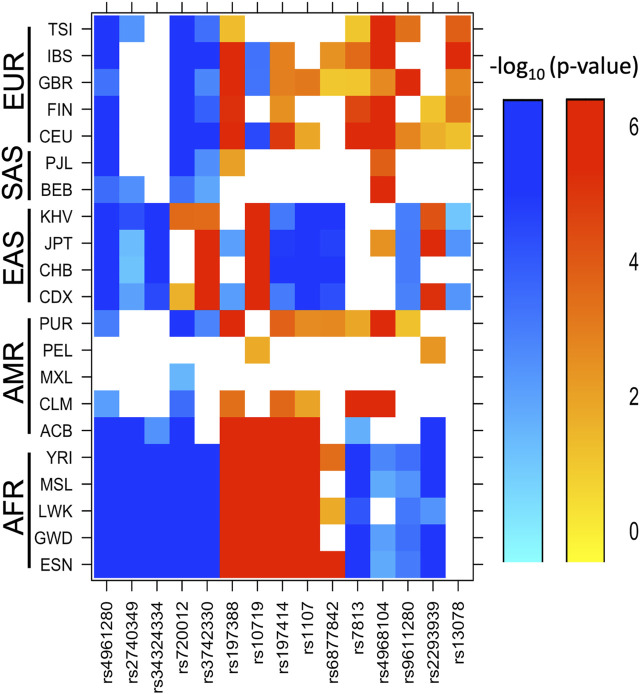
Ethnic groups with significant differences in the minor allele frequencies of SNVs with respect to Mexican individuals. The minor allele frequencies of the 15 SNVs found in the Mexican mestizo population (MEZ) in this study were compared against those reported in 21 sub-continental populations from the 1,000 Genomes database. The color gradient from light blue to dark blue indicates increasingly lower frequencies compared to MEZ (–log p-value range = 0–6). The color gradient from yellow to red indicates increasingly higher frequencies compared to MEZ (–log p-value range = 0–6). Abbreviations are ACB: African Caribbean in Barbados; BEB: Bengali in Bangladesh; CDX Chinese Dai in Xishuangbanna, China; CEU: Utah residents with Northern and Western European ancestry; CHB: Han Chinese in Beijing, China; CLM: Colombian in Medellin, Colombia; ESN: Esan in Nigeria; FIN: Finnish in Finland; GBR: British in England and Scotland; GWD: Gambian in Western Division, Gambia; IBS: Iberian populations in Spain; JPT: Japanese in Tokyo, Japan; KHV: Kinh in Ho Chi Minh City, Vietnam; LWK: Luhya in Webuye, Kenya; MSL: Mende in Sierra Leone; MXL: Mexican ancestry in Los Angeles, California; PEL: Peruvian in Lima, Peru; PJL: Punjabi in Lahore, Pakistan; PUR: Puerto Rican in Puerto Rico; TSI: Toscani in Italy and YRI: Yoruba in Ibadan, Nigeria.

After comparing our findings to those reported in the 1,000 Genomes project for 21 populations with different ethnic ancestry (Clarke et al., 2017), the MAF of SNVs rs4961280, rs2740349, rs34324334, and rs720012 in Mexican individuals was among the highest worldwide; the rs4961280 showed highest MAF across all studied populations (Figure 1; Supplementary Table S3). Similarly, the variant frequency of rs3742330 was significantly higher in Mexican individuals than in all other ethnic groups except East Asian populations. In contrast, the MAF of rs197388, rs10719, rs197414, and rs1107 in Mexican individuals was among the lowest frequencies across all analyzed populations. In addition, the frequency of variant rs6877842 in our sample was only significantly higher than the frequency in South Asian populations, whereas the frequencies of rs7813, rs4968104, rs9611280, and rs2293939 variants in Mexican individuals was intermediate between African and European populations. Finally, rs13078 showed a significant difference only with European populations (Figure 1; Supplementary Table S3). Regarding other admixed Latino American populations, the MAFs in Mexican individuals showed the greatest differences with respect to PUR and CLM.

### Worldwide distribution of minor alleles in SNVs in the miRNA biosynthesis machinery

**FIGURE 2:**
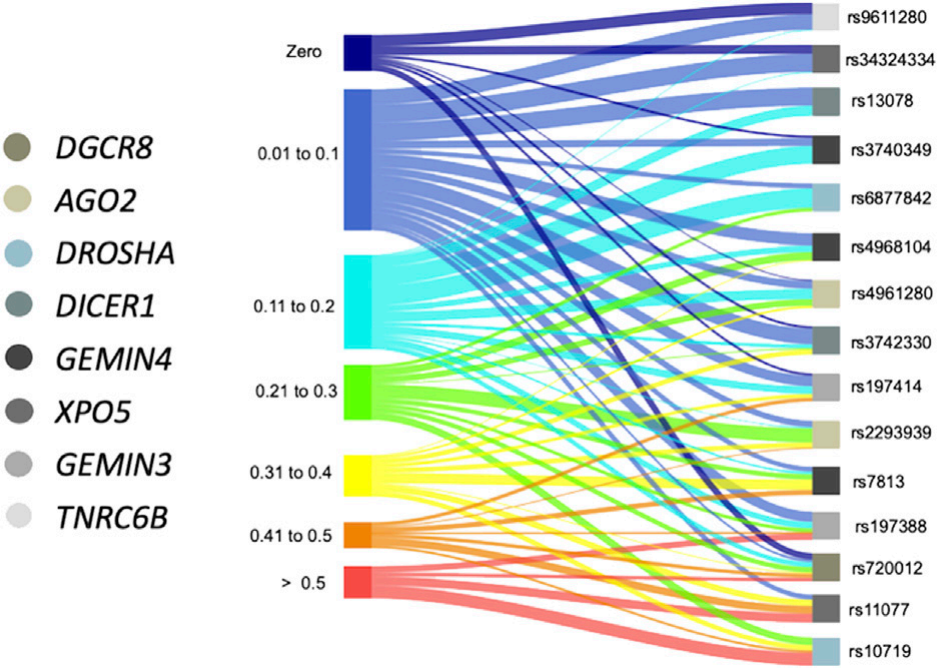
Variation in the frequency of the minor alleles in SNVs from components of the miRNA processing pathway in different ethnic groups. Sankey diagram showing the studied SNVs with connectors in different colors oriented towards the respective range of frequencies presented in 21 sub-continental populations from the 1,000 Genome project and the MEZ population from this study. Boxes in color below each SNV correspond to the gene in which each variant is located.

After analyzing the frequency distribution of the SNVs, variants rs9611280 and rs34324334 were monomorphic in 9 and 7 of the 22 populations reported in the 1,000 Genomes project, respectively, with MAFs ranging from 0.01 to 0.13 and 0.01 to 0.11 in the rest of the populations (Figure 2). In contrast, the frequency of rs11077 and rs10719 variants was extremely high in most of the ethnic groups, >0.50 in 7 and 10 of the 22 populations, respectively. The rest of the SNVs had a wide range of allele frequency distribution. For example, the frequency of the variant allele for rs13078, rs2740349, rs6877842, and rs4968104 ranged from 0.03–0.20, monomorphic-0.22, 0.01–0.25, and 0.03–0.31, respectively, whereas rs4961280, rs3742330, and rs197414, which were monomorphic in at least 1 out of the 22 populations, had MAFs as high as 0.37, 0.40, and 0.49, respectively. Similarly, the variant allele frequencies for rs2293939, rs7813, and rs197388 were as low as 0.02, 0.08, and 0.02, and as high as 0.44, 0.48, and 0.60, respectively. Importantly, variant rs720012 had the broadest distribution in its frequency worldwide, ranging from monomorphic to 0.58 (Figure 2; Supplementary Table S3).

### Population differentiation analysis

We estimated the F_ST_ value for all populations and all variants included in this work (Supplementary Figure S1). The highest F_ST_ values (F_ST_ = 0.23) were found for rs197388, followed by rs720012 (F_ST_ = 0.21) and rs197414 (F_ST_ = 0.19), whereas variants rs11077 (F_ST_ = 0.15), rs10719 (F_ST_ = 0.14), and rs3742330 (F_ST_ = 0.11) had intermediate global F_ST_ values among the studied populations. In contrast, rs4961280 (F_ST_ = 0.09), rs2293939 (F_ST_ = 0.07), rs7813 (F_ST_ = 0.06), and rs4968104 (F_ST_ = 0.05) had low global F_ST_ values among the studied population, and very low values were presented by variants rs2740349 (F_ST_ = 0.04), rs9611280 (F_ST_ = 0.03), rs6877842 (F_ST_ = 0.03), rs34324334 (F_ST_ = 0.02), and rs13078 (F_ST_ = 0.02).

**FIGURE 3:**
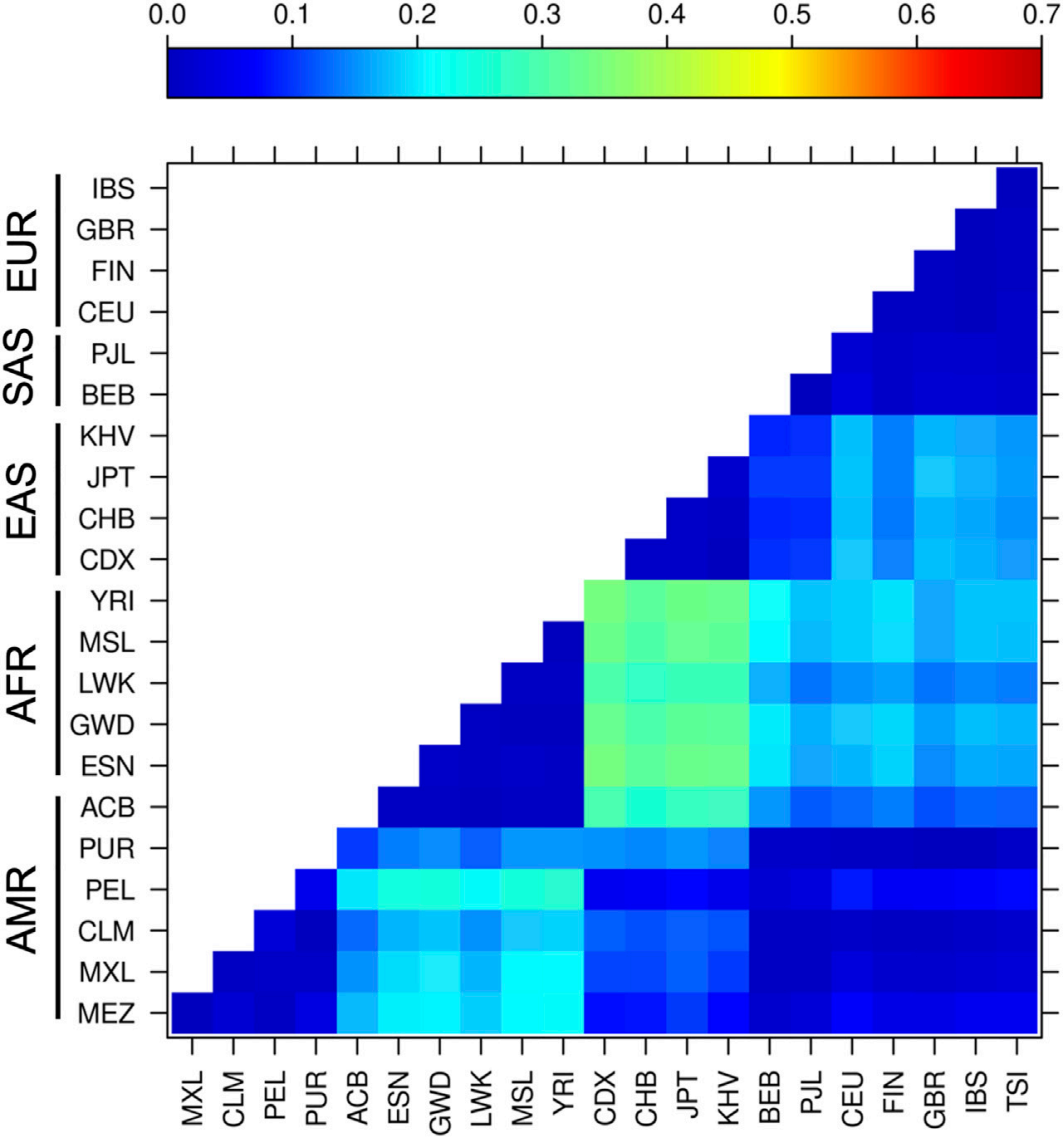
Genetic differentiation based on SNVs in components of the miRNA biosynthesis pathway. Pairwise *F*_*ST*_ analysis of all variants in 21 populations with different ancestry from the 1,000 Genomes database and MEZ population. The darkest blue indicates the lowest levels of differentiation, whereas red indicates the highest levels of differentiation. Abbreviations are the same from Figure 1.

Based on the F_ST_ analysis of the 15 variants, the African groups had the highest population differentiation compared to the other continental populations, such as the East Asian groups (F_ST_ = 0.26–0.35), South Asian group BEB (F_ST_ = 0.20–0.21), European groups CEU, FIN, and IBS (F_ST_ = 0.18–0.20), and all of the admixed Latino American groups (F_ST_ = 0.18–0.25), with exception of ACB and PUR, which have high African ancestry (Figure 3). The ACB population also exhibited important genetic differentiation with the East Asian groups (F_ST_ = 0.26–0.29). No significant differentiation was observed between the other Latino American groups and any other populations, whereas European groups exhibited slight genetic differentiation with East Asian populations (F_ST_ = 0.14–0.18).

### Correlation of gene variant frequencies with longitude and latitude

**FIGURE 4:**
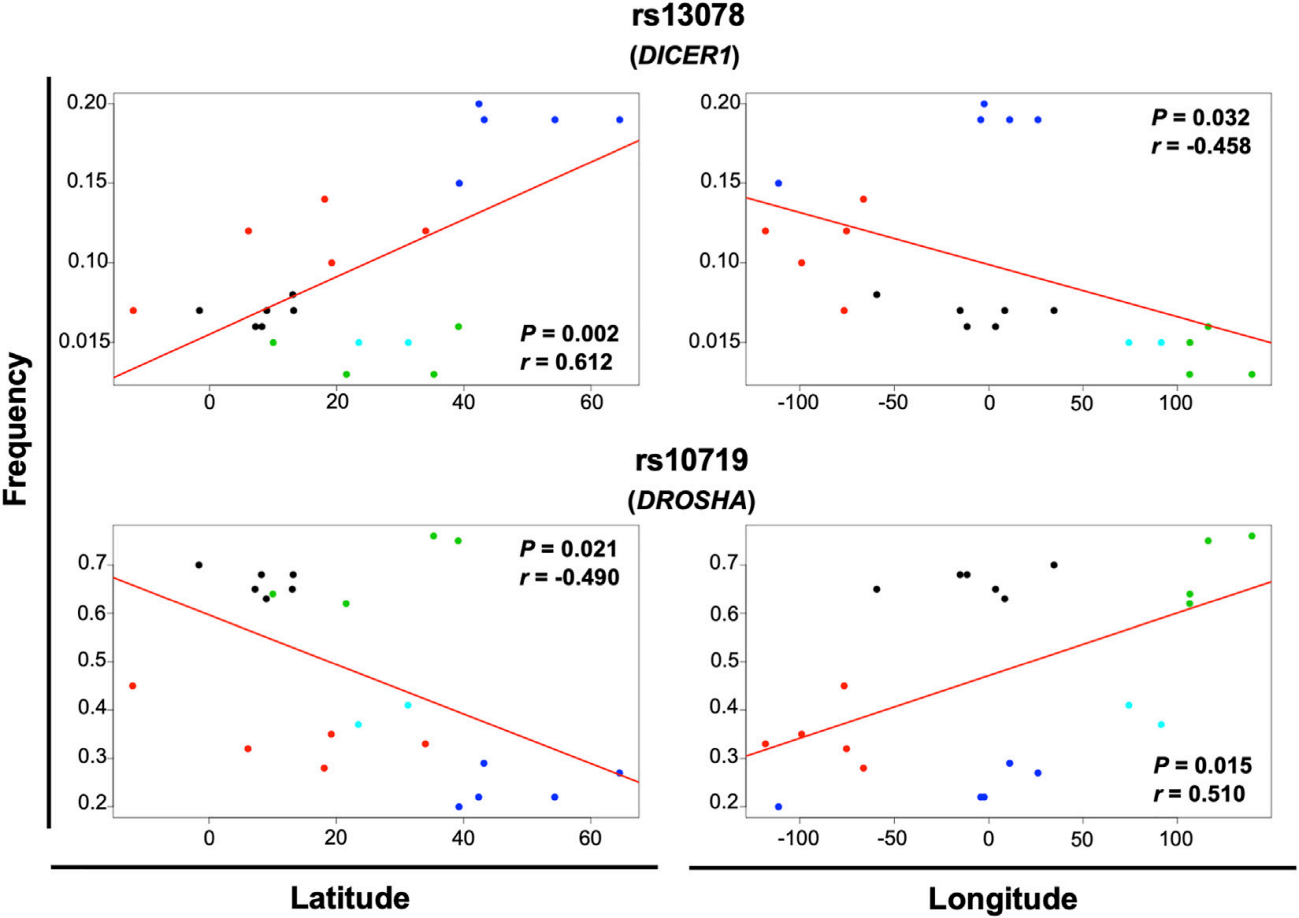
Significant correlation of the minor allele frequency of SNVs with latitude and longitude. The correlation of minor allele frequencies reported in sub-continental populations from the 1,000 Genome project and those found in this study for the MEZ population with longitude and latitude was evaluated by the Pearson’s correlation test. Gene variants with *p* < 0.05 for latitude and longitude correlation are shown in the figure. Black dots = African groups; red dots = Admixed Latino American groups; blue dots = European groups; green dots = East Asian groups; turquoise dots = South Asian groups.

**FIGURE 5:**
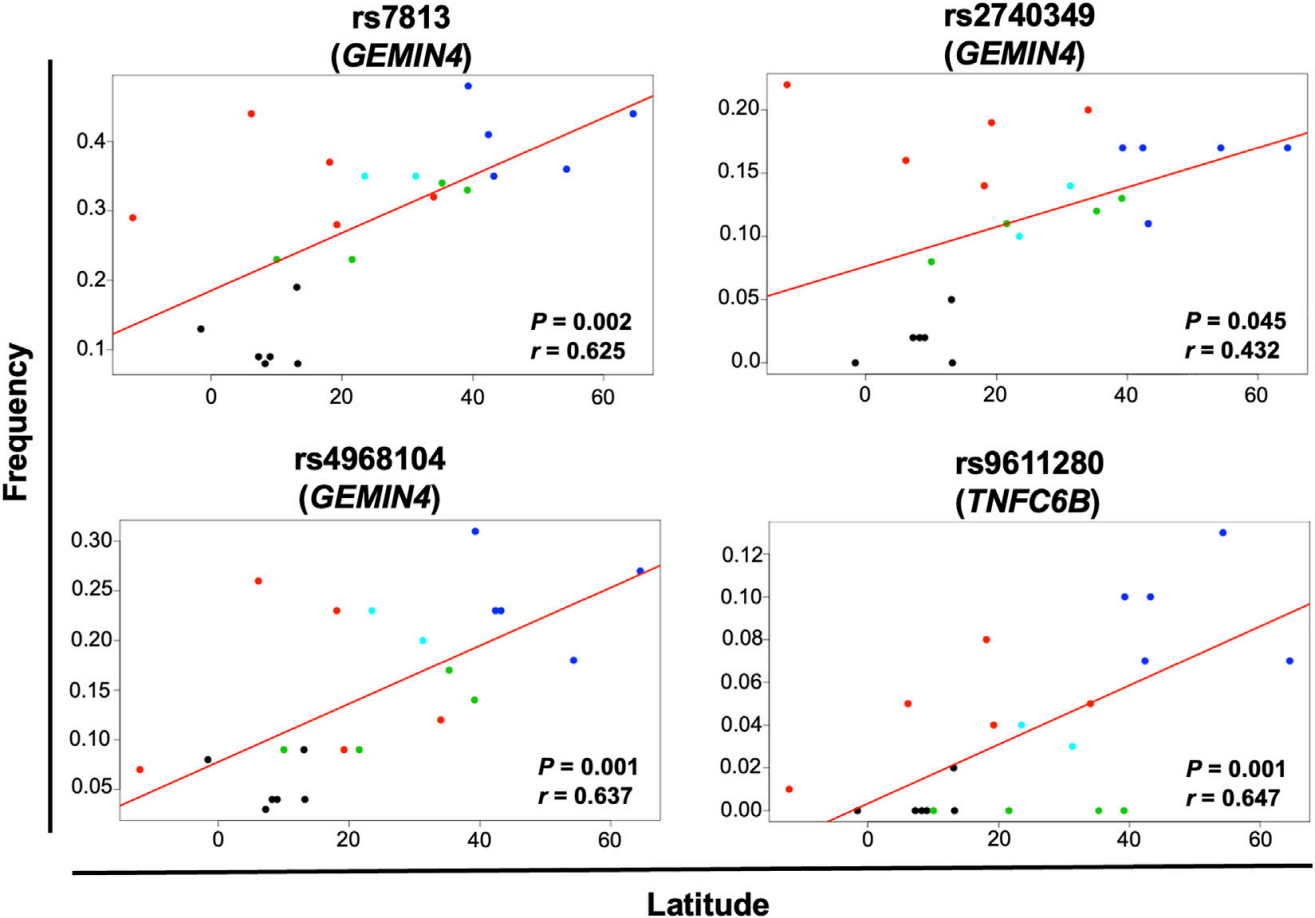
Significant correlation of the minor allele frequency of SNVs with latitude. The correlation of minor allele frequencies reported in sub-continental populations from the 1,000 Genome project and those found in this study for the MEZ population with longitude and latitude was evaluated by the Pearson’s correlation test. Gene variants with *p* < 0.05 for latitude correlation are shown in the figure. Black dots = African groups; red dots = Admixed Latino American groups; blue dots = European groups; green dots = East Asian groups; turquoise dots = South Asian groups.

**FIGURE 6:**
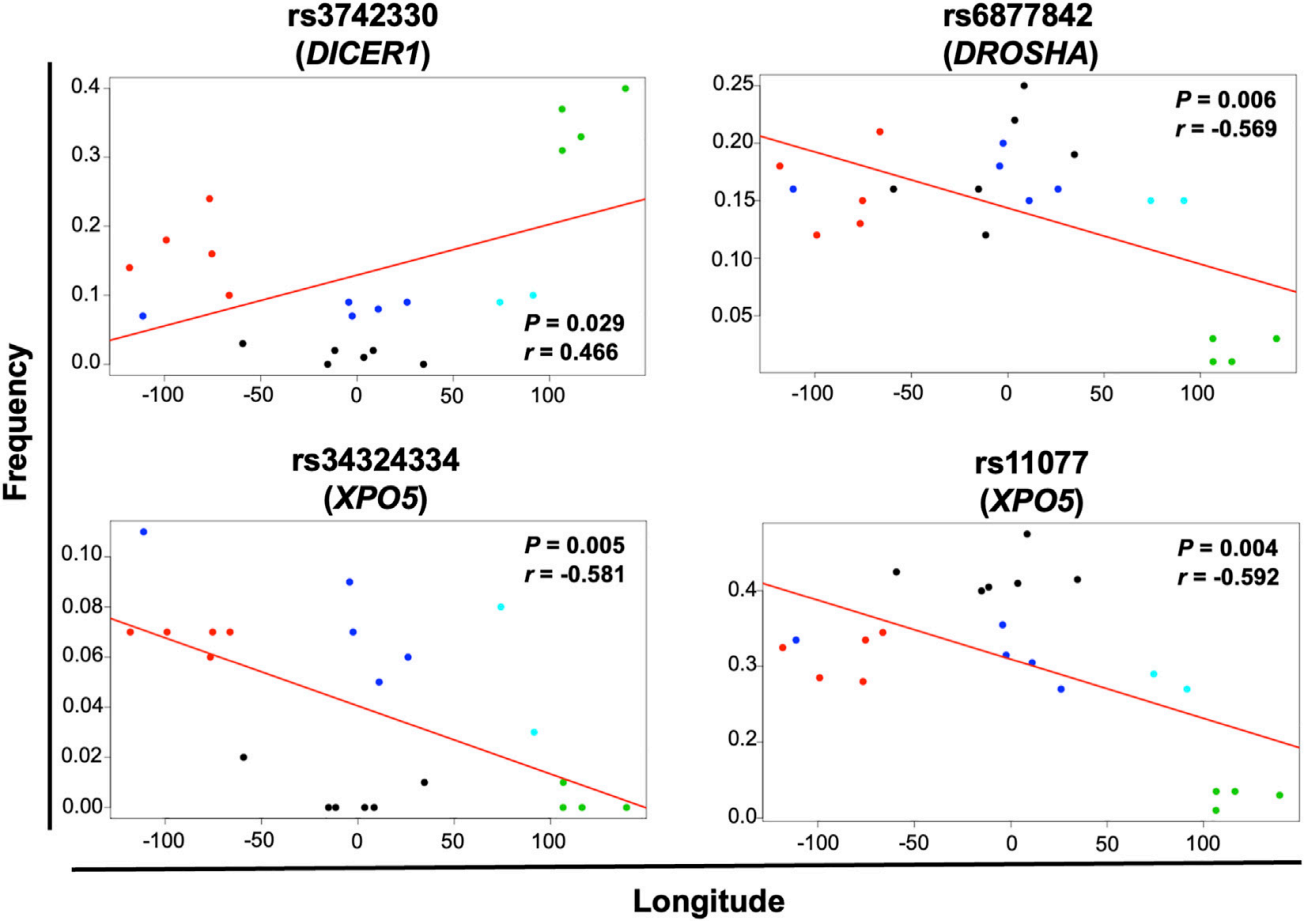
Significant correlation of the minor allele frequency of SNVs with longitude. The correlation of minor allele frequencies reported in sub-continental populations from the 1,000 Genome project and those found in this study for the MEZ population with longitude and latitude was evaluated by the Pearson’s correlation test. Gene variants with *p* < 0.05 for longitude correlation are shown in the figure. Black dots = African groups; red dots = Admixed Latino American groups; blue dots = European groups; green dots = East Asian groups; turquoise dots = South Asian groups.

Geographical factors, such as latitude and longitude, may modify the gene variant frequency. In our analysis, the SNVs rs13078 in *DICER1* and rs10719 in *DROSHA* significantly correlated with both latitude and longitude, with significant increases in the MAF from south to north (*r* = 0.612, *p* = 0.002) and significant decreases from west to east (*r* = −0.458, *p* = 0.032) in the case of rs13078, whereas the rs10719 variant significantly decreased from south to north (*r* = −0.490, *p* = 0.021) and significantly increased from west to east (*r* = 0.510, *p* = 0.015; Figure 4). The three SNVs in *GEMIN4*, rs7813, rs2740349, and rs4968104, as well as rs9611280 in *TNRC6B,* showed significant increases in MAF from south to north (*r* = 0.625, *p* = 0.002; *r* = 0.432, *p* = 0.045; *r* = 0.637, *p* = 0.001; and *r* = 0.647, *p* = 0.001, respectively; Figure 5). In addition, the frequency of variant rs3742330 in *DICER1* significantly increased from west to east (*r* = 0.466; *p* = 0.029), whereas the MAF of rs6877842 in *DROSHA* and the two variants in *XP05,* rs34324334 and rs11077, significantly decreased from west to east (*r* = −0.569, *p* = 0.006; *r* = −0.581, *p* = 0.005; *r* = −0.592, *p* = 0.004, respectively; Figure 6). SNVs rs2293939, rs4961280, rs197388, rs720012, and rs197414 did not significantly correlate with latitude or longitude.

### Distribution of gene variants in miRNA processing genes in continental populations

**FIGURE 7:**
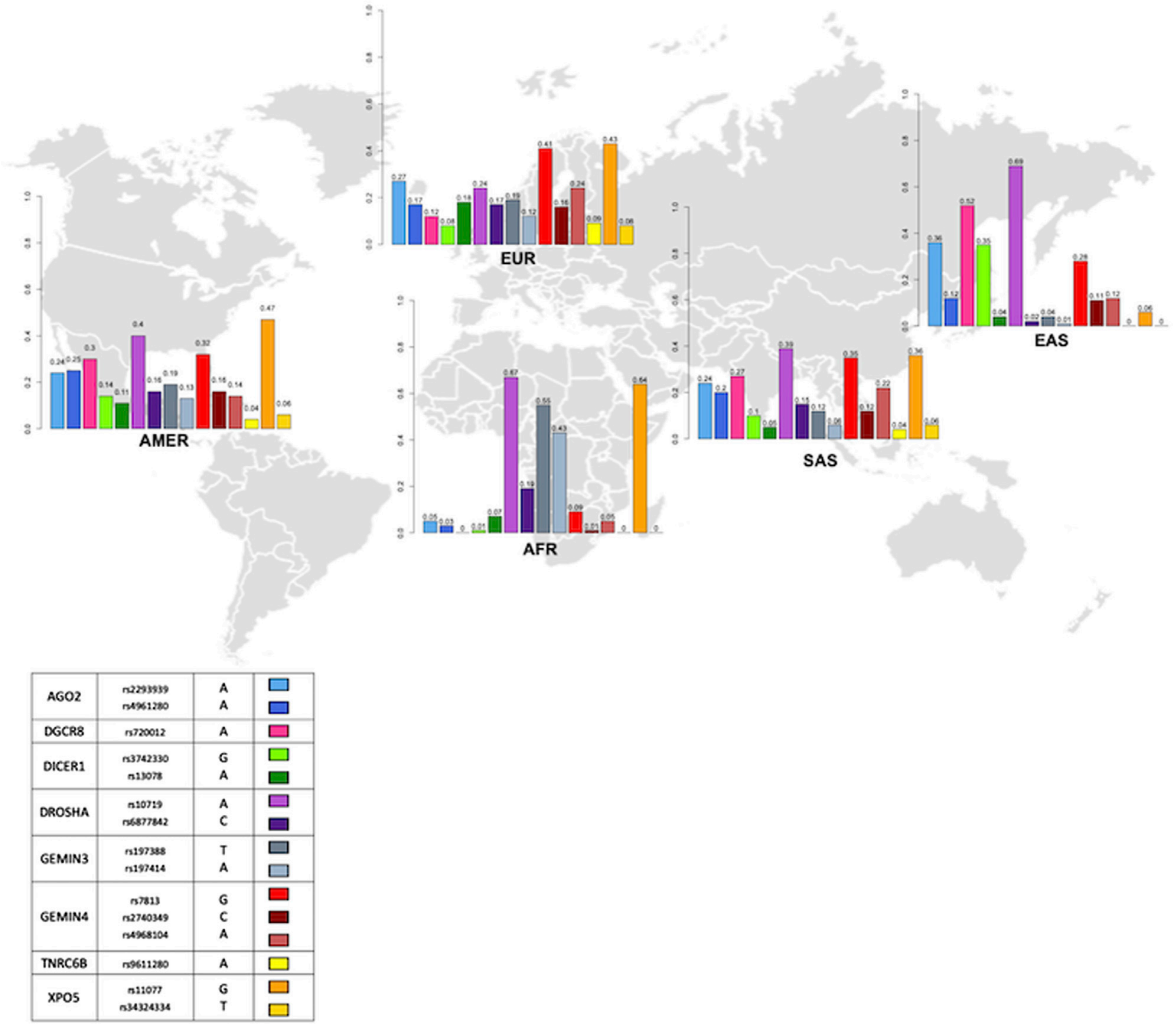
Worldwide distribution of the minor allele frequency of SNVs in components of the miRNA biosynthesis machinery in different sub-continental populations. Abbreviations in the figure are the same from Figure 1. Boxes in color denote each variant in the graphics. Numbers at the top of the bars in the graphics correspond to the minor allele frequency.

In African populations, variants rs720012 and rs9611280 were monomorphic, whereas variants rs34324334, rs3742330, rs2740349, and rs4961280 had MAFs <0.05 (Figure 7). Variants rs9611280 and rs34324334 remained monomorphic in East Asian populations and had MAFs ranging from 0.04 to 0.09 and 0.06 to 0.08, respectively, in the rest of the continental populations (Figure 7). In contrast, variants rs720012, rs4961280, rs3742330, and rs2740349 were common in populations outside of Africa, ranging from 0.08 to 0.52 (Figure 7). The highest frequencies for the variants rs720012 and rs3742330 were observed in East Asian populations (0.52 and 0.35, respectively), whereas the frequency peaks for rs4961280 and rs2740349 were found in Latino American groups (0.25 and 0.16, respectively).

SNVs rs4968104, rs13078, rs7813, and rs2293939 were common variants in African groups, with MAFs ranging from 0.05 to 0.09, whereas variants rs6877842, rs197414, rs197388, rs11077, and rs10719 were very common in these ethnic groups, with MAFs ranging from 0.19 to 0.67 (Figure 7). The rs13078 variant had a similar frequency in most of the continental populations compared to African groups (AMR = 0.11; EUR = 0.18; SAS = 0.05; EAS = 0.04 vs. AFR = 0.07), whereas variants rs4968104, rs7813, and rs2293939 significantly increased in frequency in the other continental populations (ranging from 0.04 to 0.18, 0.28 to 0.41, and 0.24 to 0.36, respectively). Finally, from the highly common variants in African populations, rs11077 and rs10719 remained high in all other continental populations except East Asian groups for rs11077 (0.06). In contrast, the MAF of SNVs rs6877842, rs197414, and rs197388 significantly decreased in all other continental populations (0.02–0.17, 0.01–0.13, and 0.04–0.19, respectively), particularly in East Asian populations (0.02, 0.01, and 0.04, respectively; Figure 7). Notably, SNV rs10719 had a MAF >0.5 in African and East Asian populations, whereas the MAF of rs197388 and rs11077 was >0.5 only in African populations, and rs720012, which was monomorphic in all African populations, had a MAF >0.5 in East Asian groups.

## Discussion

MiRNAs have become one of the most important regulatory systems in different biological events. Accordingly, SNVs located in components of the miRNA processing pathway have been associated with several human diseases. For example, SNVs in *DROSHA* have been associated with gastric cancer and susceptibility to congenital heart disease (Song et al., 2017; Borghini et al., 2021), whereas gene variants in *DICER1* occur at significantly higher allele frequencies in individuals with endometrial and hepatocellular carcinoma than in the healthy population (Wang et al., 2017; Oz et al., 2018). Based on the genetic diversification of human populations caused by migration, adaptation to local environment, and genetic drift, allele frequencies for many SNVs differ depending on the ethnicity of the population group (Lan et al., 2007; Myles et al., 2008; Gurdasani et al., 2019). In the same sense, the frequency of disease-associated alleles could also change among individuals with different ancestry.

In this study, we determined the frequency of 15 variants in eight genes from the miRNA processing pathway in Mexican individuals and compared our findings to those in 21 sub-continental populations from the 1000 Genomes project. All of the analyzed SNVs were common variants in our sample population, with rs9611280 in *TNRC6B* occurring at the lowest frequency and rs720012 in *DGCR8* being the most frequent variant. The frequencies found in our population for some of the variant alleles, such as rs10719 in *DROSHA*, rs2293939 in *AGO2*, rs7813 in *GEMIN4,* and rs9611280 in *TNRC6B,* were intermediate to the frequency previously reported in African and European groups. As the modern population in Mexico is composed of a recent and complex admixture of ancient Native American, European (mainly from Spain), and sub-Saharan Africans, this was an expected finding (Chacón-Duque et al., 2018; Aguilar-Velázquez and Rangel-Villalobos, 2021).

However, other variants showed different patterns of distribution, including rs13078 in *DICER1* and rs11077 and rs34324334 in *XP05*, which showed significant differences from either European or African populations but not both. Moreover, the minor allele of rs4961280 in *AGO2* had the highest frequency worldwide in our population, whereas the MAFs of rs2740349 in *GEMIN4* and rs720012 in *DGCR8* in Mexican individuals were among the top five in all analyzed populations. In contrast, the MAFs of rs6877842 in *DROSHA*, rs197414 and rs197388 in *GEMIN3,* and rs4968104 in *GEMIN4* were among the lowest worldwide. This could indicate the presence of geographic or climate factors modifying the frequency of the derived allele in these SNVs.

Examples of derived alleles enriched in human populations by adaptation to geography, climate conditions, and lifestyle include the lactase persistence allele in the Fula population from Western Eurasia (Schaschl et al., 2022), variants rs4766578 and rs847892 in *ALDH2*, which are associated in European individuals with resistance to consumption of high levels of alcohol (Hodgson et al., 2014), and the protective variant in the Duffy blood group gene, which provides resistance to malaria in sub-Saharan Africans (Pierron et al., 2018; Reynolds et al., 2019). In the case of admixed Latino American populations, variant alleles in *IL1R1* and *MUC1*, important regulators of the adaptive immune response, occurred at significantly higher frequencies in indigenous individuals from the southeastern region of the United States and from the central region of Mexico, respectively, compared to individuals with European ancestry (Ávila-Arcos et al., 2020). In addition, variants in genes associated with lipid metabolism, such as *APOA5*, *ABCG5,* and *ABCA1,* have strong signals of positive selection in the Mexican indigenous population (Villarreal-Molina et al., 2008; Acuña-Alonzo et al., 2010; Lindo et al., 2018). Moreover, variant alleles in *MGAM*, a gene related to starch digestion, have also been found to be enriched in Sud-American individuals compared to African and European populations (Clark et al., 2003).

As expected, the 15 variants had a high range of distribution in the different sub-continental populations evaluated. For example, rs720012 was monomorphic in all of the African populations but had a MAF ranging from 0.44 to 0.58 in East Asian populations. Similarly, the variant alleles of rs4961280 and rs3742330, which were absent in at least one African group, had frequencies ranging from 0.24 to 0.37 in admixed Latino American groups and 0.31 to 0.40 in East Asian individuals. In contrast, the variant alleles of rs9611280 and rs34324334 were monomorphic or occurred at low frequency in African and East Asian populations, with a frequency up to 0.08 and 0.11, respectively, in the rest of the analyzed sub-continental populations. Notably, rs720012, rs10719, rs197388, and rs11077 had MAFs >0.5, mainly in East Asian and African populations, suggesting the presence of selection forces in the worldwide distribution of these variants.

The functional effect of a SNV depends on its location in the structure of the gene. In this sense, the SNV rs10719 located in the 3′UTR of *DROSHA* disrupts miR-27b binding site leading to an overexpression of *DROSHA* transcript (Wen et al., 2018). Likewise, the SNV rs11077, located in the 3′UTR of *XP05*, is associated with an alteration in the stability of the mRNA, suggesting the regulation of this gene by miRNAs (Ding et al., 2013). In other examples of functional effects, rs9611280 missense variant in *TNRC6B* gene has been shown to affect the splicing of the mRNA (Martin-Guerrero et al., 2015), whereas rs4961280, located in the promoter region of *AGO2* was found to upregulate the expression of the gene in prostate cancer patients (Nikolić et al., 2017).

Based on pairwise F_ST_ analysis from the 15 analyzed variants, African populations had the highest levels of genetic differentiation with respect to all other sub-continental populations. Variants rs720012 in *DGCR*8 (FST = 0.21) and rs197388 (FST = 0.23) and rs197414 (FST = 0.19) in GE*MIN3* exhibited signals of a directional selective process according to Clark et al. (2003), who proposed values of F_ST_ > 0.18 as being suggestive of a selective process in human populations. These data suggest the functional importance of these variants and genes in the miRNA processing pathway.

The SNV rs720012 in *DGCR8* has been previously associated with non-muscle-invasive bladder cancer, tuberculosis susceptibility, and increased risk of pre-eclampsia (Ke et al., 2013; Cheng et al., 2018; Huang et al., 2019b). The rs197388 variant in *GEMIN3* has been associated with primary open-angle glaucoma in the Polish population (Molasy et al., 2018), idiopathic azoospermia in a Turkish population (Ozlem et al., 2017), and increased risk of oropharyngeal squamous cell carcinoma (Chen et al., 2016). The rs197414 variant has been associated with increased risk of bladder and esophageal cancer (Ye et al., 2008; Yang et al., 2008). The SNV rs720012 is located in the 3′UTR region of *DGCR8,* whereas rs197388 is located in the promoter region of *GEMIN3* and rs197414 is a missense variant. Although these variants have been associated with different diseases, no functional effects have been described previously.

Another important finding in our study was the significant correlation between the MAFs of rs13078 in *DICER1* and rs10719 in *DROSHA* with the geographical coordinates of latitude and longitude. The rs13078 variant allele has been associated with a decreased risk of developing type 2 diabetes and an increased risk of gestational hypertension and laryngeal cancer (Osuch-Wojcikiewicz et al., 2015; Wen et al., 2019; Huang et al., 2019a). Germinal and somatic mutations in *DICER1* have also been associated with a rare genetic cancer prone disease called pleuropulmonary blastoma familial tumor susceptibility syndrome, or DICER1 syndrome (González et al., 2022). Variant rs10719 has been associated with increased susceptibility to malignant diseases, such as colorectal cancer and gastric carcinoma (Cho et al., 2015; Li et al., 2017). This variant has also been found to be associated with metabolic diseases, including pre-eclampsia susceptibility, primary hypertension, and ischemic stroke (Kim et al., 2018; Zhang et al., 2017; Rezaei et al., 2018).

Taken together, our data suggest that the worldwide distribution of the frequency of SNVs located in components of the miRNA processing pathway has been shaped by different adaptive forces. As all of the variants analyzed in this study have been associated with genetic risk to human diseases, populations with different ancestry would present different susceptibility to specific illnesses. Our data also indicate the importance of studying admixed populations to fully understand the genetic architecture of complex human diseases.

## Data Availability

The original contributions presented in the study are included in the article/Supplementary Materials, further inquiries can be directed to the corresponding author.
