# Supplementary material for: Single nucleotide variants in microRNA biosynthesis genes in Mexican individuals
Source: Front Genet. 2023 Mar 2;14:1022912. doi: 10.3389/fgene.2023.1022912 (PMC10037310; doi:10.3389/fgene.2023.1022912)
Supplement: Supplementary file 2 [file Table1.docx]

**Supplementary Table 1.** Continental populations and subpopulations from the 1000 Genomes Project.

| Continental Population | Subpopulations | (N) |
| --- | --- | --- |
| African  (AFR) | Esan in Nigeria (**ESN**) | 99 |
|  | Gambian in Western Division, The Gambia (**GWD**) | 113 |
|  | Luhya in Webuye, Kenya (**LWK**) | 99 |
|  | Mende in Sierra Leone (**MSL**) | 85 |
|  | Yoruba in Ibadan, Nigeria (**YRI**) | 108 |
| Admixed Latino American (AMR) | African Caribbean in Barbados (**ACB**) | 96 |
|  | Colombian in Medellin, Colombia (**CLM**) | 94 |
|  | Mexican Ancestry in Los Angeles, California (**MXL**) | 64 |
|  | Peruvian in Lima, Peru (**PEL**) | 85 |
|  | Puerto Rican in Puerto Rico (**PUR**) | 104 |
| East Asian (EAS) | Chinese Dai in Xishuangbanna, China (**CDX**) | 93 |
|  | Han Chinese in Beijing, China (**CHB**) | 103 |
|  | Japanese in Tokio, Japan (**JPT**) | 104 |
|  | Kinh in Ho Chi Minh City, Vietnam (**KHV**) | 99 |
| European  (EUR) | Utah residents with Northern and Western European ancestry (**CEU**) | 99 |
|  | Finnish in Finland (**FIN**) | 99 |
|  | British in England and Scotland (**GBR**) | 91 |
|  | Iberian populations in Spain (**IBS**) | 107 |
|  | Toscani in Italy (**TSI**) | 107 |
| South Asian (SAS) | Bengali in Bangladesh (**BEB**) | 86 |
|  | Punjabi in Lahore, Pakistan (**PJL**) | 96 |
