# Supplementary material for: Single nucleotide variants in microRNA biosynthesis genes in Mexican individuals
Source: Front Genet. 2023 Mar 2;14:1022912. doi: 10.3389/fgene.2023.1022912 (PMC10037310; doi:10.3389/fgene.2023.1022912)
Supplement: Supplementary file 3 [file Presentation1.pptx]

## Slide 1
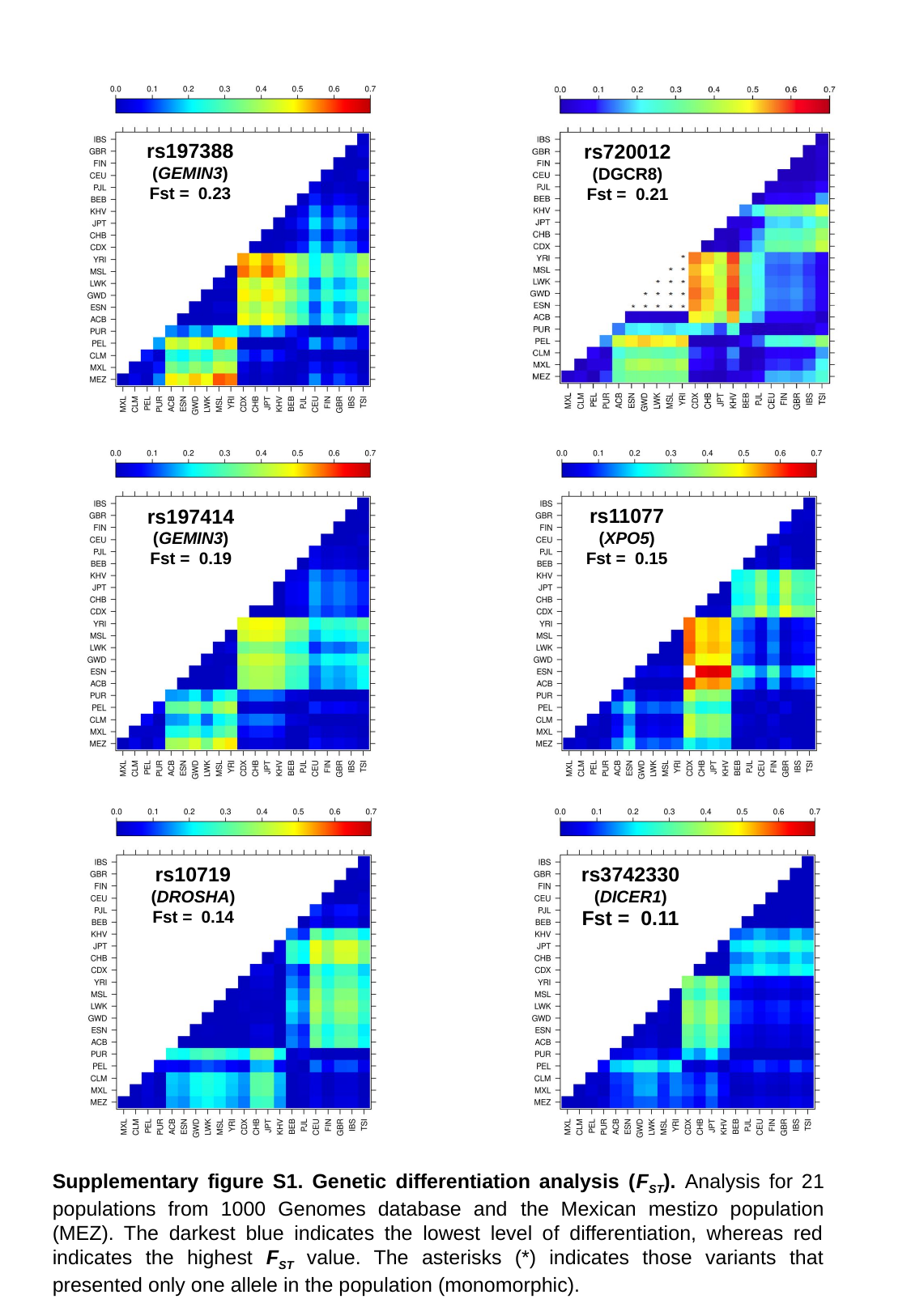

rs720012
(DGCR8)
Fst = 0.21
rs197388
(GEMIN3)
Fst = 0.23
rs197414
(GEMIN3)
Fst = 0.19
rs11077
(XPO5)
Fst = 0.15
rs10719
(DROSHA)
Fst = 0.14
rs3742330 (DICER1)
Fst = 0.11
Supplementary figure S1. Genetic differentiation analysis (FST). Analysis for 21 populations from 1000 Genomes database and the Mexican mestizo population (MEZ). The darkest blue indicates the lowest level of differentiation, whereas red indicates the highest FST value. The asterisks (*) indicates those variants that presented only one allele in the population (monomorphic).

## Slide 2
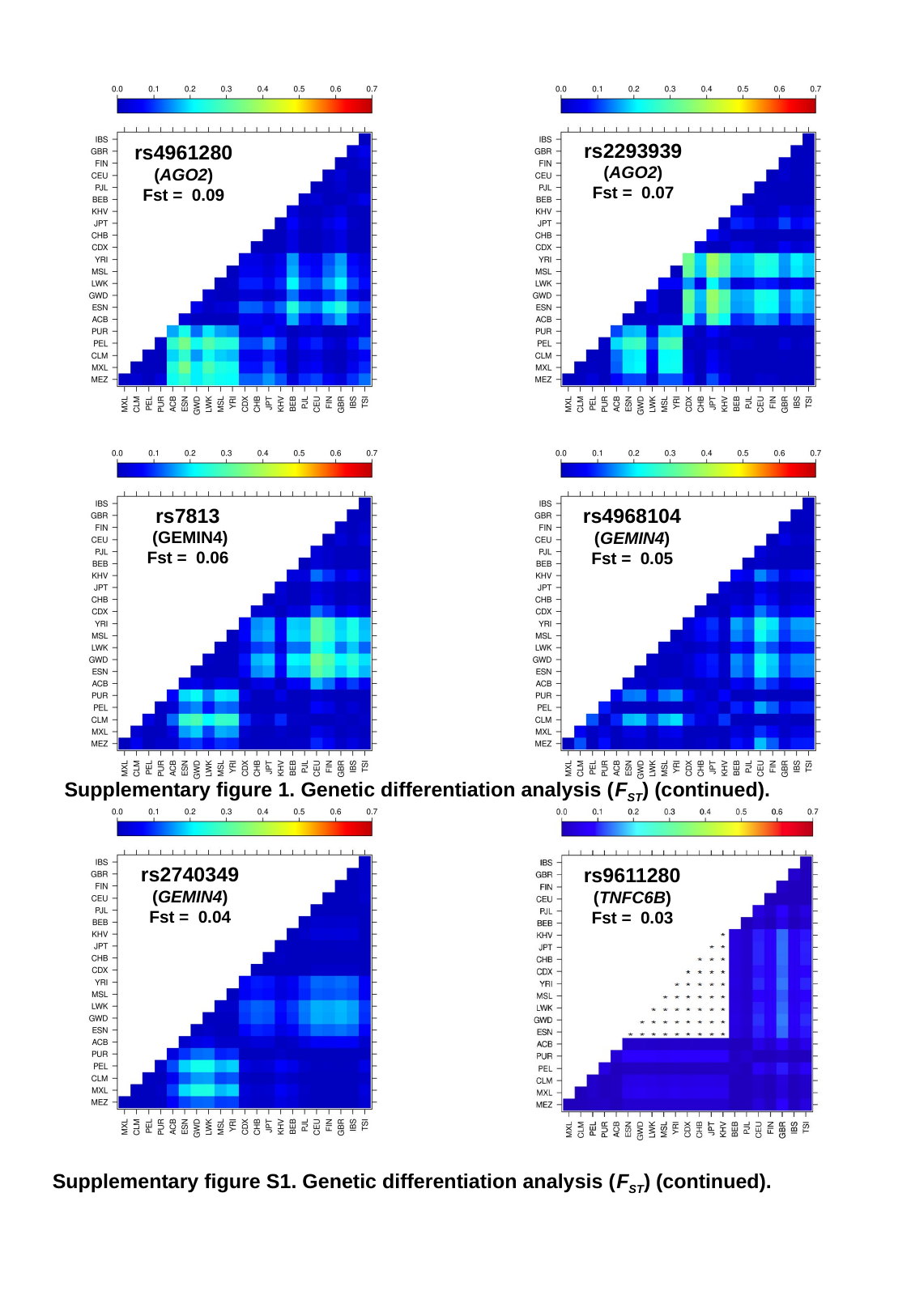

rs4961280
(AGO2)
Fst = 0.09
rs2293939
(AGO2)
Fst = 0.07
rs7813
 (GEMIN4)
Fst = 0.06
rs4968104
(GEMIN4)
Fst = 0.05
Supplementary figure 1. Genetic differentiation analysis (FST) (continued).
rs9611280
(TNFC6B)
Fst = 0.03
rs2740349
(GEMIN4)
Fst = 0.04
Supplementary figure S1. Genetic differentiation analysis (FST) (continued).

## Slide 3
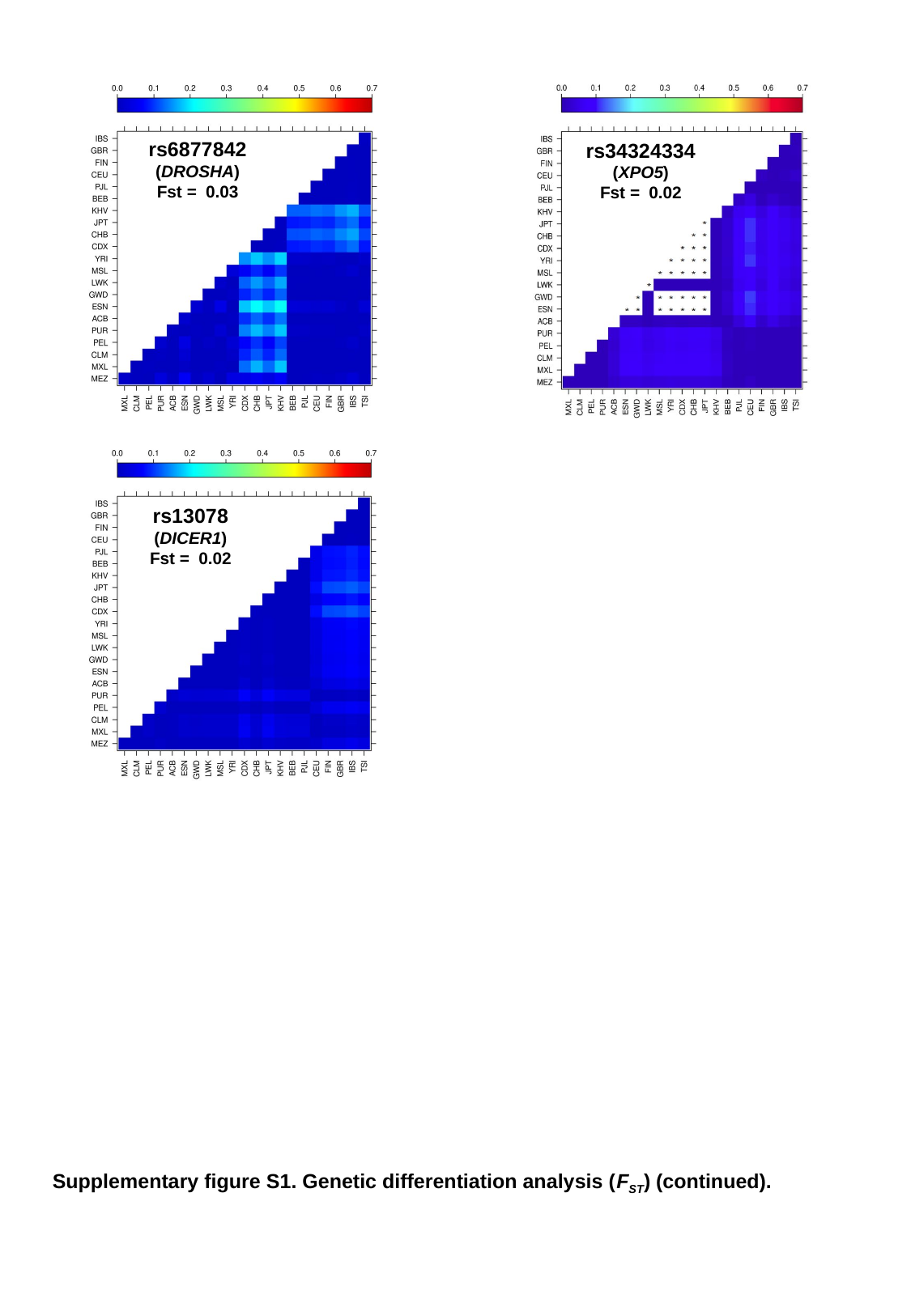

rs34324334
(XPO5)
Fst = 0.02
rs6877842
(DROSHA)
Fst = 0.03
rs13078
(DICER1)
Fst = 0.02
Supplementary figure S1. Genetic differentiation analysis (FST) (continued).
