# Supplementary material for: Single nucleotide variants in microRNA biosynthesis genes in Mexican individuals
Source: Front Genet. 2023 Mar 2;14:1022912. doi: 10.3389/fgene.2023.1022912 (PMC10037310; doi:10.3389/fgene.2023.1022912)
Supplement: Supplementary file 4 [file Table2.docx]

Supplementary Table S2. Genotypic frequency of the analyzed SNVs.

|  |  | rs10719 |  |
| --- | --- | --- | --- |
|  |  |  |  |
| Genotype | GG | GA | AA |
| Individuals | 44 | 183 | 163 |
| Frequency | 0.11 | 0.47 | 0.42 |
|  |  |  |  |
|  |  | **rs6877842** |  |
| Genotype | GG | GC | CC |
| Individuals | 303 | 88 | 3 |
| Frequency | 0.77 | 0.22 | 0.01 |
|  |  |  |  |
|  |  | **rs720012** |  |
| Genotype | AA | AG | GG |
| Individuals | 82 | 184 | 126 |
| Frequency | 0.21 | 0.47 | 0.32 |
|  |  |  |  |
|  |  | **rs11077** |  |
| Genotype | TT | GT | TT |
| Individuals | 156 | 168 | 54 |
| Frequency | 0.41 | 0.44 | 0.14 |
|  |  |  |  |
|  |  | **rs34324334** |  |
| Genotype | TT | CT | CC |
| Individuals | 1 | 49 | 339 |
| Frequency | 0.0 | 0.13 | 0.87 |
|  |  |  |  |
|  |  | **rs2293939** |  |
| Genotype | AA | GA | GG |
| Individuals | 24 | 128 | 242 |
| Frequency | 0.06 | 0.32 | 0.61 |
|  |  |  |  |
|  |  | **rs4961280** |  |
| Genotype | AA | **CA** | CC |
| Individuals | 55 | 174 | 158 |
| Frequency | 0.14 | 0.45 | 0.41 |
|  |  |  |  |
|  |  | **rs3742330** |  |
| Genotype | AA | **GA** | GG |
| Individuals | 246 | 114 | 12 |
| Frequency | 0.66 | 0.31 | 0.03 |
|  |  |  |  |
|  |  | **rs13078** |  |
| Genotype | AA | TA | TT |
| Individuals | 3 | 60 | 286 |
| Frequency | 0.01 | 0.17 | 0.82 |
|  |  |  |  |
| Genotype |  | **rs197388** |  |
| Individuals | AA | **TA** | TT |
| Frequency | 340 | 52 | 3 |
|  | 0.86 | 0.13 | 0.01 |
|  |  |  |  |
|  |  | **rs197414** |  |
| Genotype | AA | **CA** | CC |
| Individuals | 4 | 34 | 350 |
| Frequency | 0.01 | 0.09 | 0.90 |
|  |  |  |  |
|  |  | **rs7813** |  |
| Genotype | AA | **GA** | GG |
| Individuals | 201 | 162 | 28 |
| Frequency | 0.51 | 0.41 | 0.07 |
|  |  |  |  |
|  |  | **rs2740349** |  |
| Genotype | TT | **CT** | CC |
| Individuals | 260 | 118 | 17 |
| Frequency | 0.66 | 0.3 | 0.04 |
|  |  |  |  |
|  |  | **rs4968104** |  |
| Genotype | AA | **TA** | TT |
| Individuals | 5 | 64 | 325 |
| Frequency | 0.01 | 0.16 | 0.82 |
|  |  |  |  |
|  |  | **rs9611280** |  |
| Genotype | AA | **AG** | **GG** |
| Individuals | 2 | 27 | 369 |
| Frequency | 0.01 | 0.06 | 0.93 |
