# Supplementary material for: Single nucleotide variants in microRNA biosynthesis genes in Mexican individuals
Source: Front Genet. 2023 Mar 2;14:1022912. doi: 10.3389/fgene.2023.1022912 (PMC10037310; doi:10.3389/fgene.2023.1022912)
Supplement: Supplementary file 5 [file Table4.docx]

**Supplementary figure S1. Genetic differentiation analysis (*F_ST_*). Analysis for 21 populations from 1000 Genomes database and the Mexican mestizo population (MEZ). T**he darkest blue indicates the lowest level of differentiation, whereas red indicates the highest ***F_ST_*** value. The asterisks (*) indicates those variants that presented only one allele **in the population** (monomorphic).


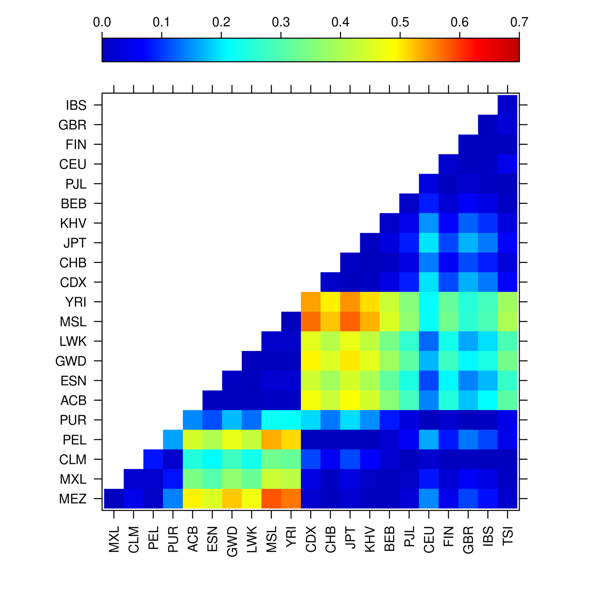


**rs197388**

**(*GEMIN3*)**

**Fst = 0.23**


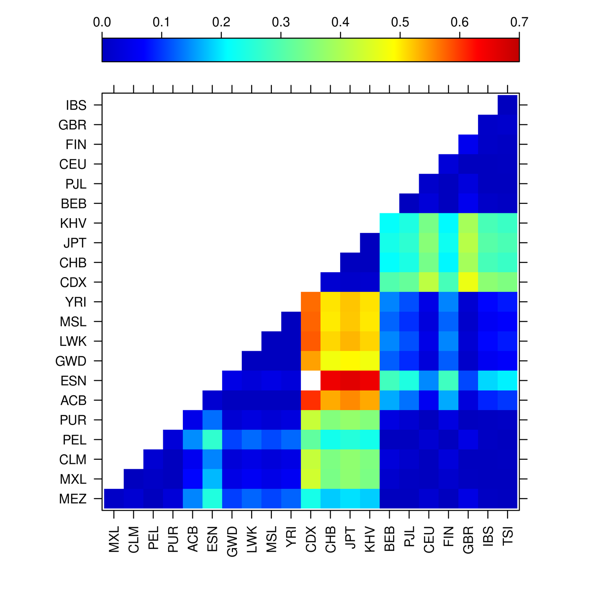


**rs11077**

**(*XPO5*)**

**Fst = 0.15**


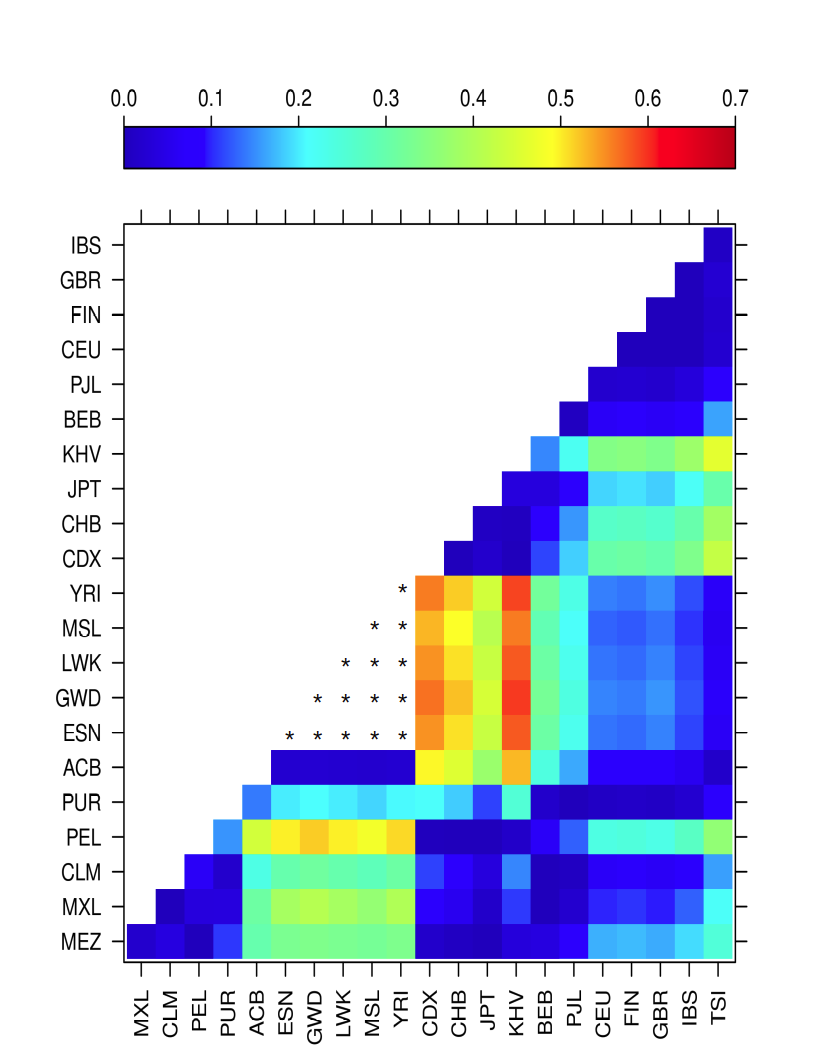


**rs720012**

**(DGCR8)**

**Fst = 0.21**


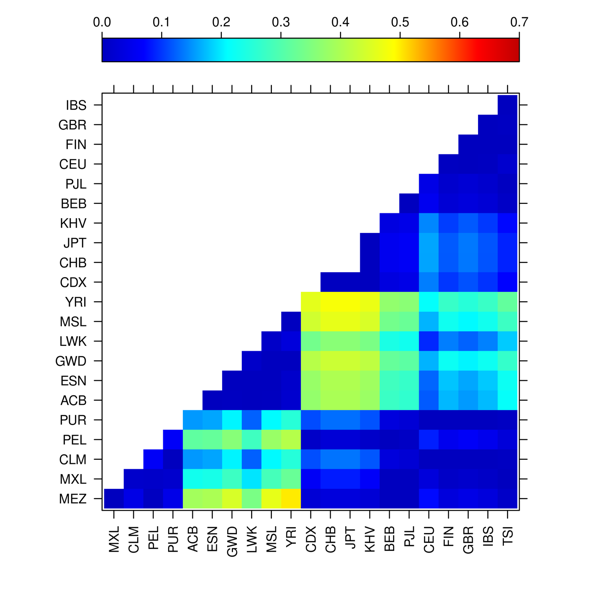


**rs197414**

**(*GEMIN3*)**

**Fst = 0.19**


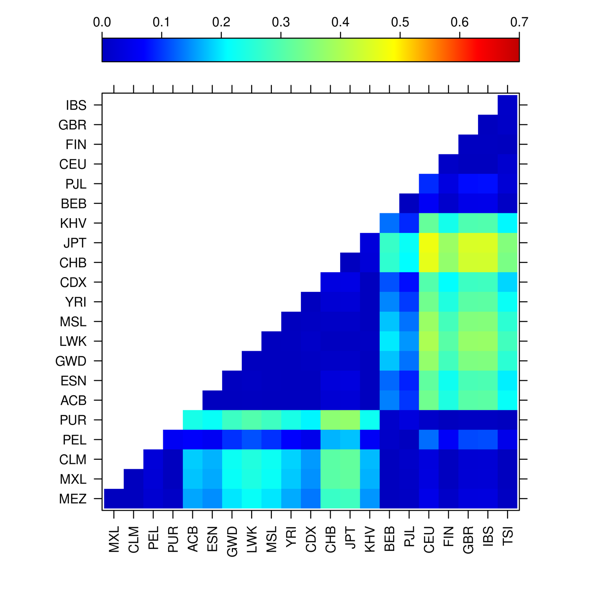


**rs10719**

**(*DROSHA*)**

**Fst = 0.14**


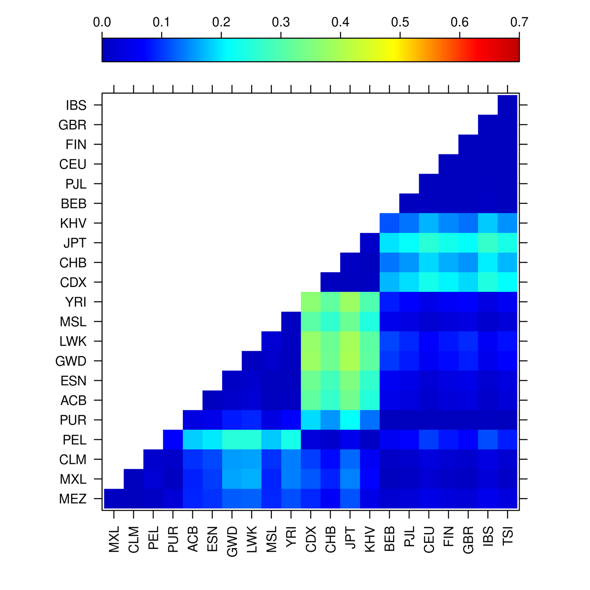


**rs3742330 (*DICER1*)**

**Fst = 0.11**


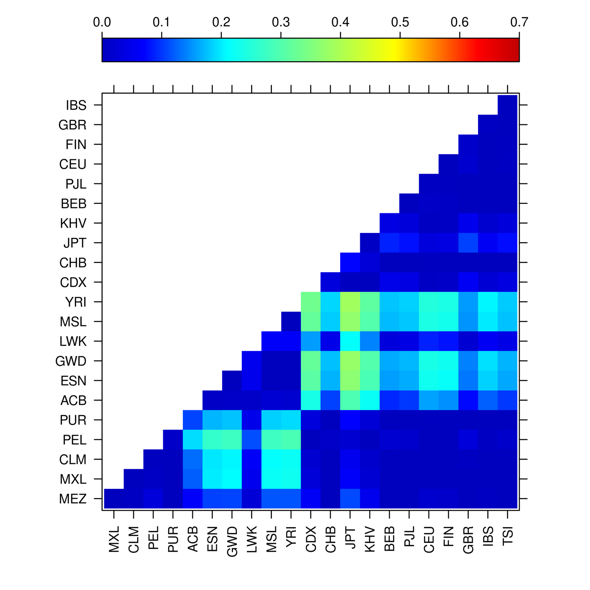


**rs2293939**

**(*AGO2*)**

**Fst = 0.07**


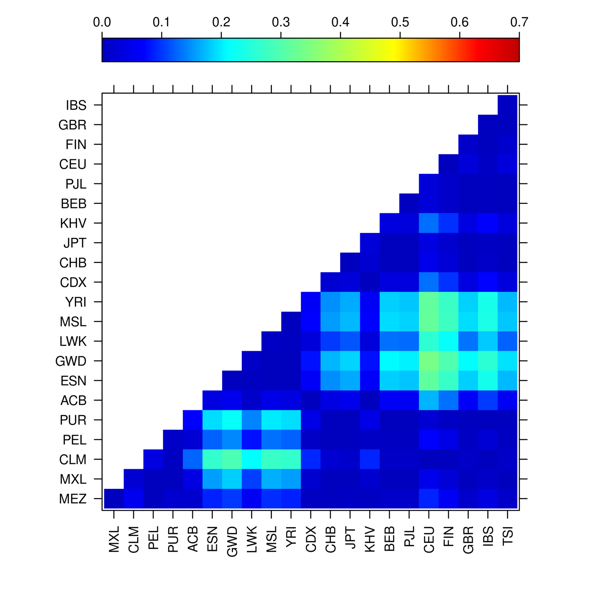


**rs7813**

**(GEMIN4)**

**Fst = 0.06**


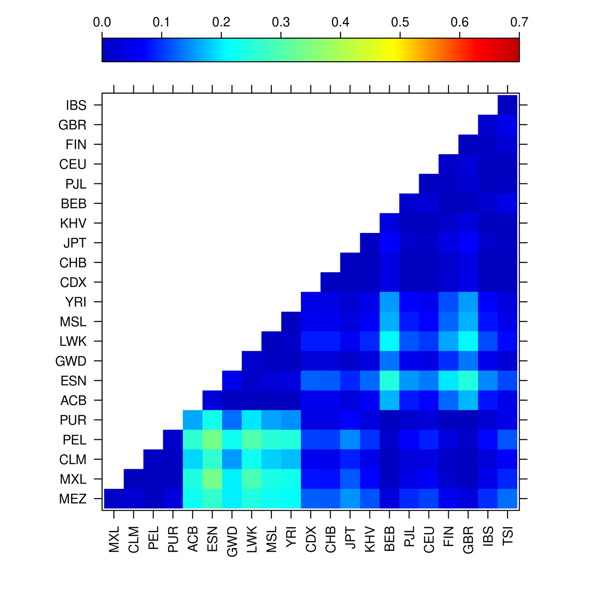


**rs4961280**

**(*AGO2*)**

**Fst = 0.09**


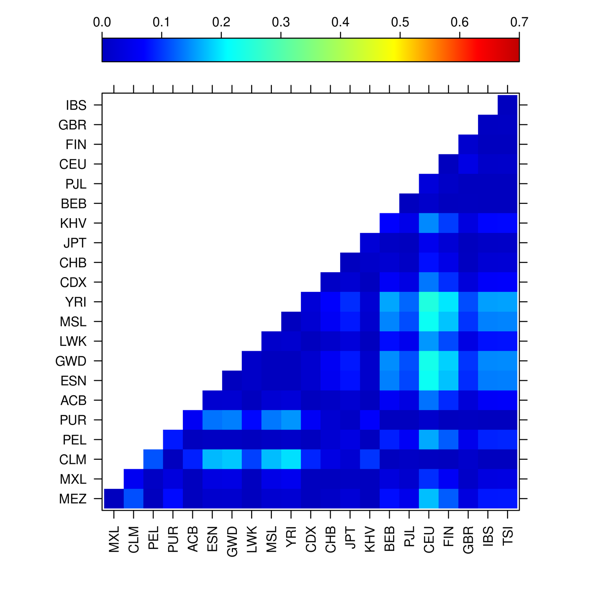


**rs4968104**

**(*GEMIN4*)**

**Fst = 0.05**


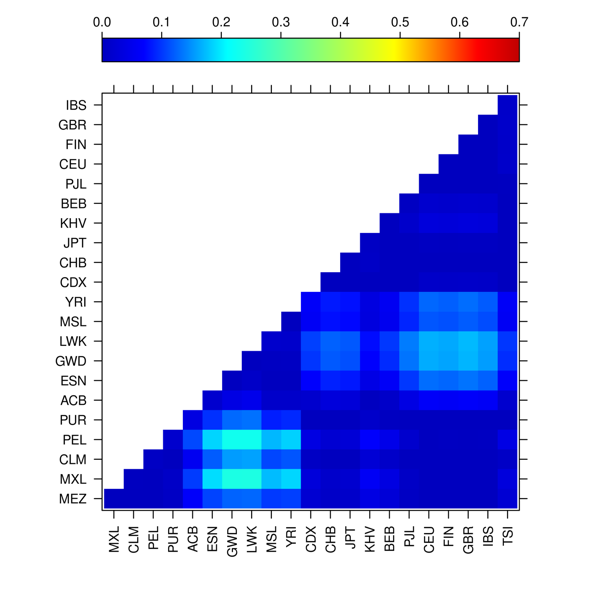


**rs2740349**

**(*GEMIN4*)**

**Fst = 0.04**


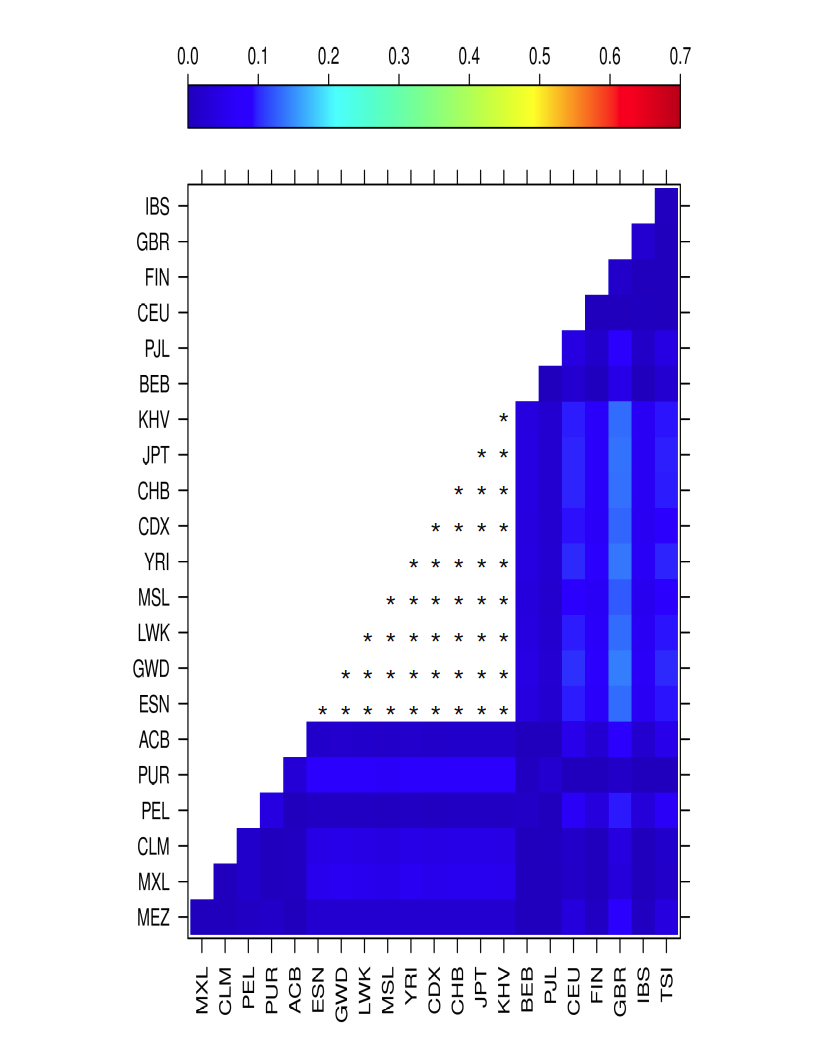


**rs9611280**

**(*TNFC6B*)**

**Fst = 0.03**

**Supplementary figure S1. Genetic differentiation analysis (*F_ST_*) (continued).**


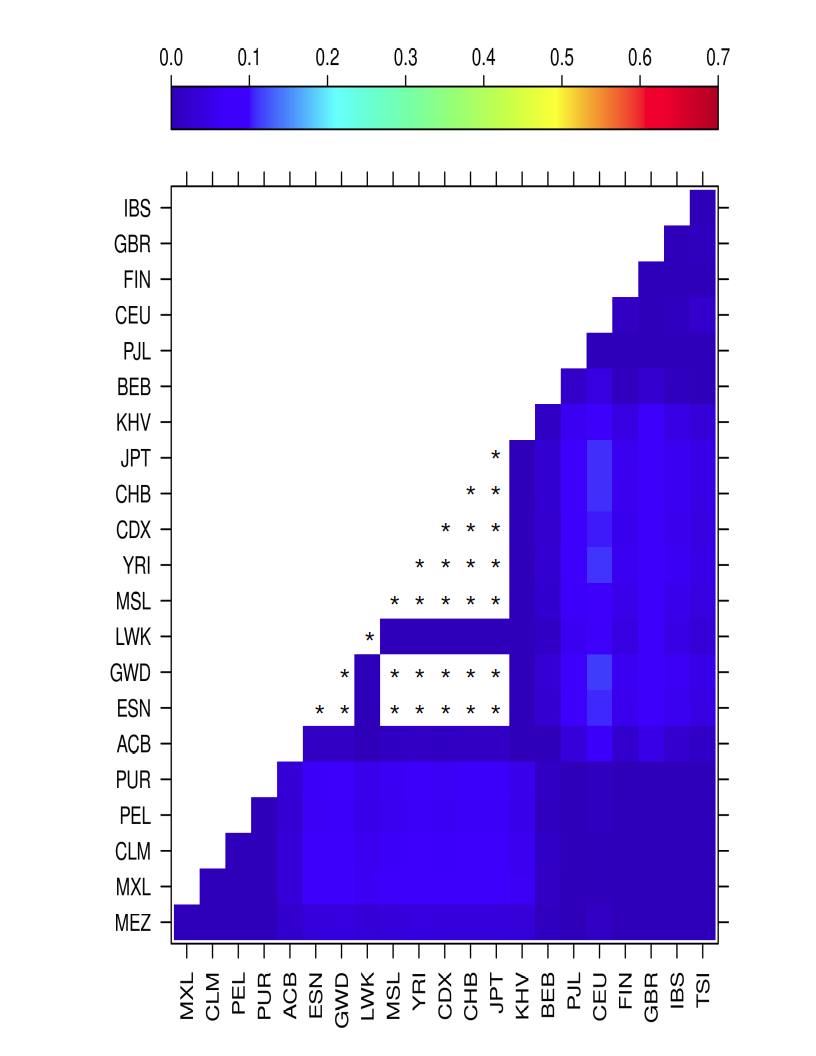


**rs34324334**

**(*XPO5*)**

**Fst = 0.02**


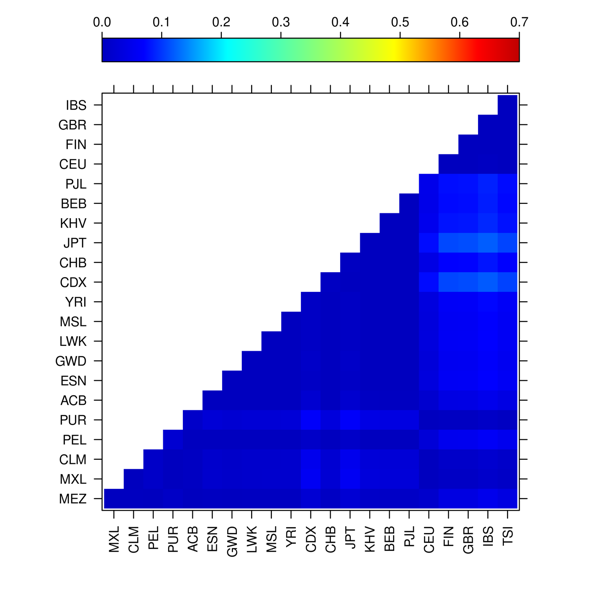


**rs13078**

**(*DICER1*)**

**Fst = 0.02**


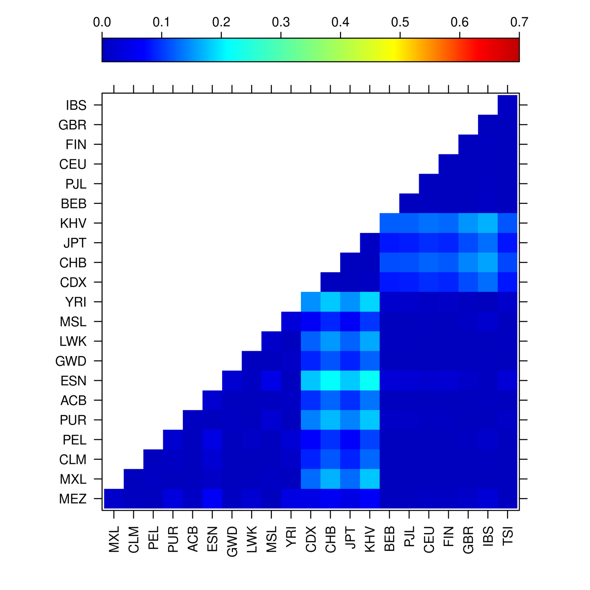


**rs6877842**

**(*DROSHA*)**

**Fst = 0.03**

**Supplementary figure S1. Genetic differentiation analysis (*F_ST_*) (continued).**
